# Infection of Dendritic Cells With *Mycobacterium avium* subspecies *hominissuis* Exhibits a Functionally Tolerogenic Phenotype in Response to Toll-Like Receptor Agonists *via* IL-10/Cox2/PGE2/EP2 Axis

**DOI:** 10.3389/fmicb.2019.01795

**Published:** 2019-08-07

**Authors:** Woo Sik Kim, Joo-Heon Yoon, Min-Kyoung Shin, Sung Jae Shin

**Affiliations:** ^1^Department of Microbiology and Institute for Immunology and Immunological Diseases, Brain Korea 21 PLUS Project for Medical Science, Yonsei University College of Medicine, Seoul, South Korea; ^2^Advanced Radiation Technology Institute, Korea Atomic Energy Research Institute, Jeongeup, South Korea; ^3^Department of Otorhinolaryngology, Yonsei University College of Medicine, Seoul, South Korea; ^4^Department of Microbiology, College of Medicine, Institute of Health Sciences, Gyeongsang National University, Jinju, South Korea

**Keywords:** *Mycobacterium avium* subspecies *hominissuis*, dendritic cells, interleukin-10, toll-like receptor4, Cox-2/PGE_2_ signaling, EP2 receptor

## Abstract

*Mycobacterium avium* subspecies *hominissuis* (MAH) is the most common agent causing nontuberculous mycobacterial disease in humans. It mainly causes chronic and slowly progressive pulmonary disease (PD), which requires a long-term treatment and allows opportunistic co-infection by common pulmonary pathogens such as *Pseudomonas aeruginosa*, *Staphylococcus aureus*, and *Aspergillus* spp., thereby resulting in alteration of host immune response. In the present study, we investigated the phenotypical and functional alterations of dendritic cells (DCs), a bridge antigen-presenting cell between innate and adaptive immunity, following MAH infection in response to various toll-like receptor (TLR) agonists mimicking co-infection conditions, along with subsequent T cell response. Interestingly, MAH-infected DCs produced interleukin (IL)-10 significantly and decreased the level of IL-12p70 in response to Poly I:C and LPS, although not so in response to Pam3CSK4, imiquimod, or CpG oligodeoxynucleotide, thereby indicating that the TLR3 and TLR4 agonists functionally altered MAH-infected DCs toward a tolerogenic phenotype. Moreover, IL-10-producing tolerogenic DCs were remarkably induced by MAH and *P. aeruginosa* co-infection. To precisely elucidate how these TLR agonists induce tolerogenic DCs upon MAH infection, we sought to clarify the major mechanisms involved, using LPS, which caused the greatest increase in IL-10 production by the TLR agonists. Increased IL-10 stimulated the creation of tolerogenic DCs by significantly reducing MHC class II expression and MHC class II-antigen presentation, eventually inhibiting CD4^+^ T cell proliferation, along with decreased IFN-γ and IL-2. The tolerogenic phenotypes of MAH/LPS-treated DCs were restored by anti-IL-10 neutralization, validating the induction of tolerogenicity by IL-10. Interestingly, IL-10-producing-tolerogenic DCs were observed after infection with live MAH, rather than with inactivated or dead MAH. In addition, TLR2^−/−^ and TLR4^−/−^ DCs confirmed the association of IL-10 production with TLR2 and TLR4 signaling; IL-10 production synergistically increased when both TLR4 and TLR2 were involved. Expression of Cox2 and PGE2 increased along with IL-10 while that of IL-10 was inhibited by their selective inhibitors celecoxib and anti-EP2 antibody, respectively. Thus, the tolerogenic phenotypes of MAH/LPS-treated DCs were proven to be induced by Cox-2/PGE2-dependent EP2 signaling as the main mechanism. These findings may provide important clues that the tolerogenic cascade in MAH-infected DCs induced by TLR 4 signaling can alter host immune response.

## Introduction

*Mycobacterium avium* subspecies *hominissuis* (MAH), which belongs to the *M. avium* complex (MAC), is emerging as an important pathogen in pulmonary diseases (PD) caused by nontuberculous mycobacterial (NTM) infection in humans, despite the decreasing incidence of tuberculosis globally (Prevots and Marras, 2015; Meier et al., 2017). The prevalence of chronic lung diseases, such as chronic obstructive pulmonary disease (COPD), cystic fibrosis (CF), and bronchiectasis, and HIV infection have increased, and the use of immunosuppressive medication has increased, resulting in increased frequency of NTM infection (Thomson, 2010; Bonaiti et al., 2015). Recently, patients with NTM-PD were seen to be frequently co-infected with other NTM species or microorganisms (Wickremasinghe et al., 2005; Kunst et al., 2006; Fujita et al., 2014; Bonaiti et al., 2015). Especially, *Pseudomonas aeruginosa* (*P. aeruginosa*) has been reported to range from 27 to 52% and is the most frequent co-infection with NTM (Wickremasinghe et al., 2005; Zoumot et al., 2014; Bonaiti et al., 2015). According to Wickremasinghe et al. (2005), *P. aeruginosa* was isolated in 52% of patients co-infected with NTM in a retrospective analysis of 100 patients with bronchiectasis (Wickremasinghe et al., 2005). *Staphylococcus aureus* (28%), *Haemophilus influenzae* (12%), *Candida albicans* (8%), *Aspergillus fumigatus* (4%), and *Stenotrophomonas maltophilia* (4%) were the other frequent co-pathogens in the same study (Wickremasinghe et al., 2005; Bonaiti et al., 2015). Fujita et al. (2014) identified pathogenic co-infection of non-MAC pathogens, isolated from the sputum samples of 275 patients with MAC-PD between 2001 and 2013; 45.1, 14.9, and 40% patients with MAC-PD showed chronic, intermittent, and no co-infection, respectively (Fujita et al., 2014). The main co-infecting microorganisms from the above findings were *S. aureus*, *P. aeruginosa*, and *Aspergillus* spp. in higher order (Fujita et al., 2014). In particular, these three major microorganisms chronically infected patients with MAC-PD, accounting for 71.9% of *S. aureus* infection, 77.8% of *P. aeruginosa* infection, and 62.1% of *Aspergillus* spp. infection (Fujita et al., 2014). *P. aeruginosa* was reported to be more frequently isolated during MAC treatment (75%) or after MAC sputum conversion (93.1%) than during MAC-positive sputum culture (25.7%) (Fujita et al., 2014). This implies that *P. aeruginosa* may be able to infect intermittently, but the most *P. aeruginosa* infection can affect chronically patients with MAC (Fujita et al., 2014). Chronic co-infection of *P. aeruginosa* is known to be associated with a wide range of lesions in the lower lobe of lung and can affect lung function and disease severity (Fujita et al., 2014; Hsieh et al., 2018).

While cellular mechanisms for mycobacterial infection, such as pathogenesis and immune response, have been relatively well studied, the studies have been limited to single mycobacterial species. Therefore, the phenotypic and functional alterations of immune cells are not yet clearly understood in MAH and other microorganism co-infection. Dendritic cells (DCs), as professional antigen presenting cells linking innate and acquired immunity, prime T cells after migration to secondary lymph node, and induce subsequent T cell responses (Jiao et al., 2002; Kim et al., 2017). In other words, the initial immune response that MAH and other microorganisms encounter may take place in DCs, which ultimately determine the direction of T cell response (Kim et al., 2017). Toll-like receptors (TLRs) expressed on dendritic cell surfaces play an important role in recognizing specific bacterial, viral, and fungal structural molecules known as pathogen-associated molecular patterns (PAMPs) and initiating innate immune responses (Mogensen, 2009; Feng et al., 2019). TLRs are also known to play a pivotal role in mycobacterial infections (Mahla et al., 2013). TLR2 has been reported to be a major receptor that plays an important role in the recognition of major mycobacterial surface components, such as lipoprotein and lipoarabinomannan, and in the control of *M. avium* infection (Yoshimura et al., 1999; Jones et al., 2001). Other TLRs have also been shown to recognize *M. avium* and induce specific immune responses. TLR4, which is known as a receptor for Gram-negative bacterial LPS, is involved in signal transduction *via* the heat-labile cell wall proteins of mycobacteria (Quesniaux et al., 2004). TLR6 and TLR9 have also been reported to recognize lipoproteins and mycobacterial DNA and induce a host inflammatory response against mycobacteria (Bafica et al., 2005; Marinho et al., 2013). Additionally, various TLR agonists are also used as agents to mimic the mechanism of immune-cell stimulation by microbial signals (Feng et al., 2019); for example, Pam3CSK4 (mimicking bacterial lipopeptide; agonist of TLR1/2) (Werling et al., 2009), polyinosinic:polycytidylic acid (poly I:C, a synthetic double-stranded RNA (dsRNA); agonist of TLR3) (Matsumoto and Seya, 2008), lipopolysaccharide (LPS, a cell-wall component of Gram-negative bacteria; agonist of TLR4) (Bryant et al., 2010), imiquimod (a synthetic imidazoquinolone amine, which has potent immune response modifier activity; agonist of TLR7) (Sauder, 2000), and CpG oligodeoxynucleotide (CpG ODN, short single-stranded synthetic DNA molecule; agonist of TLR9) (Bauer et al., 2001).

In the present study, we aimed to analyze the immune response of DCs by stimulating with various TLR agonists to mimic co-infection, ultimately to identify T cell responses in MAH-infected DCs. We described a specific immune response of MAH-infected DCs in response to the various TLR stimuli and explored a major mechanism driving it. Our results provide important clues for the identification of complex immune mechanisms activated by MAH co-infection.

## Materials and Methods

### Cell Culture

Bone marrow cells isolated from WT, TLR2 knockout (TLR2^−/−^), and TLR4^−/−^ mice (C57BL/6 backgrounds, Jackson Laboratory, Bar Harbor, ME, USA) at 6–7 weeks of age was cultured at 37°C in the presence of 5% CO_2_ using RPMI medium (GIBCO, Carlsbad, CA, USA) supplemented with 20 ng/ml GM-CSF (JW Creagene, Gyeonggi, Korea), 10% fetal bovine serum (FBS, Lonza, Basel, Switzerland), and 1% antibiotics (penicillin/streptomycin, Lonza). On day eight, the CD11c^+^ cell fraction was labeled with a bead-conjugated anti-CD11c monoclonal antibody (mAb; Miltenyi Biotec, San Diego, CA, USA) and separated by sequential passages using LS columns (Miltenyi Biotec). Purity of the selected bone marrow-derived DCs (BMDCs) CD11c^+^ fractions was >95%.

### Cytokine Analysis in Culture Supernatants

To investigate the cytokine pattern induced in MAH-infected BMDCs by treatment with TLR ligands, selected BMDCs were infected with MAH 104 [multiplicity of infection (MOI) of 1] for 2 h prior to treatment with Pam3CSK4 (TLR2 ligand), poly I:C (TLR3 ligand), LPS from *Escherichia coli* O111:B4 (TLR4 ligand), imiquimod (R837, TLR7 ligand), and ODN1826 (TLR9 ligand) for 24 h at 37°C. To investigate the cytokine pattern induced in BMDCs exposed to TLR ligands by infection with MAH 104, BMDCs were treated with each TLR ligand (TLR2, 3, 4, 7, and 9) for 2 h prior to infection with MAH 104 for 24 h at 37°C. Finally, to investigate the cytokine pattern induced by co-treatment with MAH 104 and TLR stimulators (TLR ligands, *P. aeruginosa* PA01, or *P. aeruginosa* NCCP14781), BMDCs were treated with MAH 104 and TLR ligands, *P. aeruginosa* PA01 and TLR ligands, or *P. aeruginosa* NCCP14781 and TLR ligands (representative Gram-negative bacteria; MOI of 0.1, 0.5, and 1) for 24 h at 37°C. To determine cytokine concentrations, culture supernatants were analyzed using the sandwich ELISA kit (eBioscience, San Diego, CA, USA) according to the manufacturer’s instructions. All TLR ligands were obtained from Invivogen (San Diego, CA, USA).

### Cell Viability Assay

For Annexin V and PI staining, BMDCs were infected with MAH (MOI of 1) in the presence or absence of LPS (100 ng/ml) at 37°C. After 24 h, the cells were harvested, washed twice with PBS, and stained using Annexin V and PI apoptosis Detection kit (BD Bioscience, San Jose, CA, USA) according to the manufacturer’s instructions. The cells were analyzed using a LSRII flow cytometer (Becton Dickinson, San Jose, CA, USA). Under the same culture conditions for Annexin V and PI staining, Cell Counting Kit-8 (CCK-8) reagent (Dojindo Laboratories, Tabaru, Japan) was added according to the manufacturer’s instructions to each well for 1 h at 37°C. Viable cells were analyzed by the absorbance at 450 nm, using a microplate ELISA reader.

### Expression of Surface Molecules for Dendritic Cells

The cells (non-, LPS-, MAH-, MAH/LPS-treated DCs) were harvested, washed twice with PBS, and stained using anti-CD11c, anti-CD80, anti-CD86, anti-MHC-I, and anti-MHC-II mAbs for 30 min at 4°C. Thereafter, cells were washed three times with PBS, analyzed using a LSRII flow cytometer, and examined using FlowJo software. All surface antibodies were obtained from eBioscience.

### Intracellular Cytokine Staining

Stimulated cells were treated with GolgiPlug for 12 h, stained with anti-CD11c Ab for 20 min at room temperature (RT), and then fixed and permeabilized with a Cytofix/Cytoperm kit (BD Bioscience) following the manufacturer’s instructions. Next, anti-TNF-α (eBioscience), anti-IL-12p70 (eBioscience), and anti-IL-10 (eBioscience) Abs were stained with fluorescein-conjugated secondary Abs in a permeabilization buffer and analyzed using a LSRII flow cytometer.

### Antigen-Presenting Ability

To investigate the differences in peptide-MHC-I and -MHC-II complex formations in the absence and presence of MAH infection, cells were stimulated with either no treatment (non), LPS, MAH, or LPS and MAH, in the presence of 500 μg/ml OVA protein or 25 μg/ml Eα_44–76_ peptide (RLEEFAKFASFEAQGALANIAVDKANLDVMKKR; underlined sequence bound to MHC-II). OVA_257–264_ peptide (SIINFEKL) or Eα_52–68_ peptide (ASFEAQGALANIAVDKA) acts as a positive control for peptide formations of MHC-I or MHC-II, respectably. After 24 h of treatment, each cell was stained with anti-CD11c, anti-H-2Kb (SIINFEKL, eBioscience), or anti-I-Ab-Eα_52–68_ (Y-Ae; ASFEAQGALANIAVDKA, eBioscience) mAbs for 30 min at 4°C. Data were collected on LSRII and analyzed using FlowJo software. All peptides were synthesized by AbFrontier (Seoul, Korea).

### Mixed Lymphocyte Reaction

Each single-cell suspension, isolated from the spleens of OVA-specific T cell receptor transgenic mice (OT-I and OT-II mice from C57BL/6 backgrounds) and BALB/C mice were labeled with bead-conjugated anti-CD4 and anti-CD8 mAbs (Miltenyi Biotec) and separated by sequential passaging using LS columns. Selected cells were stained with CFSE (5 μM, eBioscience) at 37°C and then washed with PBS (supplemented with 2% FBS) after 15 min. Next, in the presence or absence of OVA-specific OVA_323–339_ or OVA_257–264_ treatments, the cells (non-, LPS-, MAH-, MAH/LPS-treated DCs) were co-cultured with CFSE-labeled T cells (from naïve BALB/C, OT-I, or OT-II mice) at a DC to a T cell ratio of 0.5:1. After 3 days of co-culture, cells were harvested and stained with anti-CD4 and anti-CD8 mAbs (eBioscience). Data were collected on LSRII and analyzed using the FlowJo software. Cytokines, including IFN-γ and IL-2, were analyzed from the culture supernatants using cytokine-specific ELISA kits (eBioscience), following the manufacturer’s instructions.

### Heat and Paraformaldehyde Inactivation for *Mycobacterium avium* subspecies *hominissuis*

In cell cultures with MAH, MAH were killed either by heating at 100°C for 30 min or by a 1-h incubation at RT in 4% (w/v) paraformaldehyde (PFA). Bacteria inactivated with PFA were extensively washed off with PBS. In the presence or absence of heat or PFA inactivation, the production of cytokines in non-, LPS-, MAH-, and MAH/LPS-treated DCs was analyzed using ELISA.

### Immunoblot Analyses

For the cytosolic fraction, cells were lysed with RIPA lysis buffer (Pierce, Rockford, IL, USA). The subsequent steps were carried out as previously described (Kim et al., 2013). Western blotting Abs and HRP-conjugated anti-rabbit Abs were obtained from Santa Cruz Biotechnology, Inc. (Santa Cruz, CA, USA) and Sigma-Aldrich (St. Louis, Mo, USA), respectively.

### Treatment of Pharmacological Inhibitor and PGE_2_ Receptor Blockers

Following pre-treatment with Cox-2 inhibitor (celecoxib; 1 μM) or PGE2 receptor blockers (1 μg/ml each of anti-EP2 and -EP4 mAb) for 1 h, cells were infected with MAH (MOI of 1) in the presence or absence of LPS treatment for 24 h at 37°C, and IL-10 concentration was evaluated by ELISA. Cox-2 inhibitor and PGE2 receptor blockers were obtained from Sigma-Aldrich and Abcam (Cambridge, MA, USA), respectively.

### Statistical Analysis

Levels of significance of the differences between samples were determined by Tukey’s multiple comparison test and unpaired *t*-test using a statistical software (GraphPad Prism Software, version 5; GraphPad Software, San Diego, CA, USA). We performed a normality test using Shapiro-Wilk test. At the 0.05 level, all data were significantly drawn from a normally distributed population. The data in the graphs are expressed as the means. Results were considered to be statistically significant at ^*^p < 0.05, ^**^p < 0.01, or ^***^p < 0.001.

## Results

### Toll-Like Receptor4 Agonist Stimulation of *Mycobacterium avium* subspecies *hominissuis*-Infected Dendritic Cells Induces Marked Interleukin-10 Production

**Figure 1:**
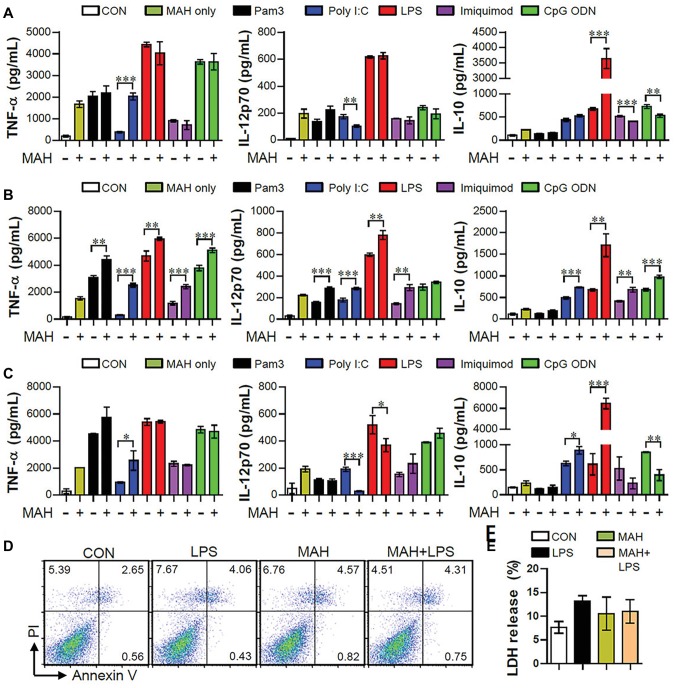
MAH-infected DCs induced high-level anti-inflammatory cytokine secretion, but no pro-inflammatory cytokine, during TLR4 agonist stimulation. **(A,B,C)** Immature DCs were treated with PBS (CON), various TLR agonists, and MAH (at a MOI of 1) under three conditions. After 24 h, culture supernatants were collected, and IL-12p70, TNF-α, and IL-10 production analyzed using ELISA. **(A)** DCs were treated with PBS (CON) and various TLR agonists after 2-h MAH infection. **(B)** DCs were treated with PBS (CON) and MAH after 2-h treatment with various TLR agonists. **(C)** DCs were treated with PBS (CON), various TLR agonists, MAH, or MAH with various TLR agonists for 24 h. **(D,E)** DCs were treated with PBS (CON), LPS, MAH, or MAH with LPS (100 ng/ml) for 24 h. **(D)** After incubation, apoptotic and necrotic cell deaths were measured by PI and Annexin V assay. **(E)** The surviving cells were quantified with CCK-8 assay. The data are expressed as the mean ± SD of 4 samples per treated condition. One representative plot out of three independent experiments is shown. ^*^p < 0.05, ^**^p < 0.01, or ^***^p < 0.001.

TLRs play key roles in host defense against various pathogens, which may contribute to the PAMP-induced signaling pathways (Mogensen, 2009). In order to study how co-infection of MAH and various pathogens affects cytokine production in DCs, we first confirmed the regulation of cytokine production (pro- and anti-inflammatory cytokines) in DCs following MAH infection for 2 h prior to stimulation with TLR agonist for 24 h (Figure 1A). Interestingly, MAH-infected DCs dramatically increased the levels of anti-inflammatory cytokine IL-10 in the presence of TLR4 agonist (LPS) stimulation, although this cytokine level was not significantly different in the presence of other TLR signaling molecules. The increased patterns of TNF-α and IL-12p70 levels were not shown. Next, we analyzed the regulation of cytokine production following TLR agonist treatment for 2 h prior to infection with MAH for 24 h (Figure 1B). TNF-α and IL-12p70 production in DCs, induced by the treatment of TLR2, 3, 4, 7, and 9 agonists, were strongly increased by MAH infection; IL-10 production in DCs, induced by the treatment of TLR3, 4, 7, and 9 agonists, was increased by MAH infection, but not in TLR2-treated DCs. Finally, we analyzed cytokine production in DCs following treatment with both MAH and TLR ligands (Figure 1C). As shown in Figure 1C, MAH-infected DCs had considerably higher levels of the anti-inflammatory cytokine IL-10 in the presence of TLR4 signaling (LPS stimulation), although this cytokine level was not significantly different in the presence of other TLR signaling molecules. TNF-α and IL-12p70 production, depicted in Figures 1A,C, showed similar patterns for treatment of all TLR agonists, although IL-10 levels strongly increased in Figure 1C (LPS treatment condition) than in Figures 1A,B. Based on these results, to investigate the role of excessive IL-10 production in DCs co-infected with MAH and TLR-stimulations, the method in Figure 1C was selected for further investigation. We also confirmed that the remarkable enhancement in IL-10 production in MAH-infected DCs exposed to LPS was not due to cytotoxicity, since there was no difference in Annexin V/PI positive cells and LDH release (Figures 1D,E). We also identified the above results using *P. aeruginosa*, a Gram-negative organism, which is reported to be a major co-infecting agent in patients with NTM-PD (Wickremasinghe et al., 2005), in order to confirm the specific immune response of MAH-infected DCs in real co-infection. *P. aeruginosa* PA01 and *P. aeruginosa* NCCP14781 were tested with the third method (followed in Figure 1C), and expression of IL-10 was explosively increased in the MAH/*P. aeruginosa*-infected DC (Supplementary Figure S1).

### *Mycobacterium avium* subspecies *hominissuis*-Infected Dendritic Cells Induce Tolerogenic Phenotypes in the Presence of Toll-Like Receptor4 Signaling

**Figure 2:**
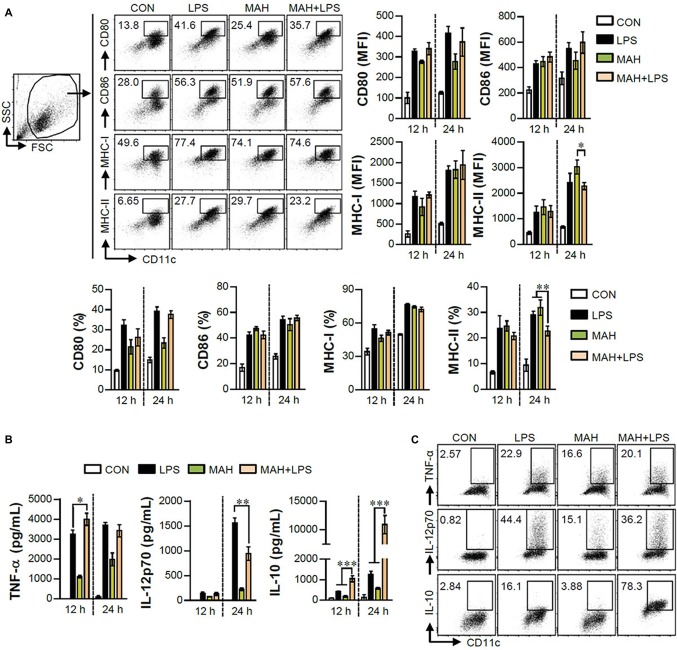
MAH infection reduced the expression of MHC class II molecules, and facilitated the production of IL-10 in presence of LPS treatment. **(A,B)** DCs were treated with PBS (CON), LPS, MAH, or LPS with MAH for the indicated times, and cells and supernatants were harvested. **(A)** Cells were stained with surface Abs (anti-CD80, anti-CD86, anti-MHC-I, and anti-MHC-II) of DCs, and analyzed by flow cytometry. DC population was drawn based on characteristic FSC and SSC patterns. Matured DCs were further gated to obtain CD11c^+^CD80^+^, CD11c^+^CD86^+^, CD11c^+^MHC-I^+^, and CD11c^+^MHC-II^+^. Dot blot for the expression of surface molecules is shown as a representative plot out of three independent experiments. Bar graphs are expressed as the mean ± SD of 3 samples per treated condition. **(B)** IL-12p70, TNF-α, and IL-10 production in the culture supernatants was analyzed by ELISA. Bar graphs are expressed as the mean ± SD of 3 samples per treated condition. **(C)** DCs were treated with PBS (CON), LPS, MAH, or LPS with MAH in presence of GolgiPlug. After 12 h, cells were fixed/permeabilized and stained with anti-TNF-α, anti-IL-12p70, and anti-IL-10 mAbs and analyzed by flow cytometry. Intracellular cytokine levels were drawn based on the CD11c^+^TNF^+^, CD11c^+^IL-12p70^+^, and CD11c^+^IL-10^+^ cells. One representative plot, out of four independent experiments, is shown. ^*^p < 0.05, ^**^p < 0.01, or ^***^p < 0.001.

**Figure 3:**
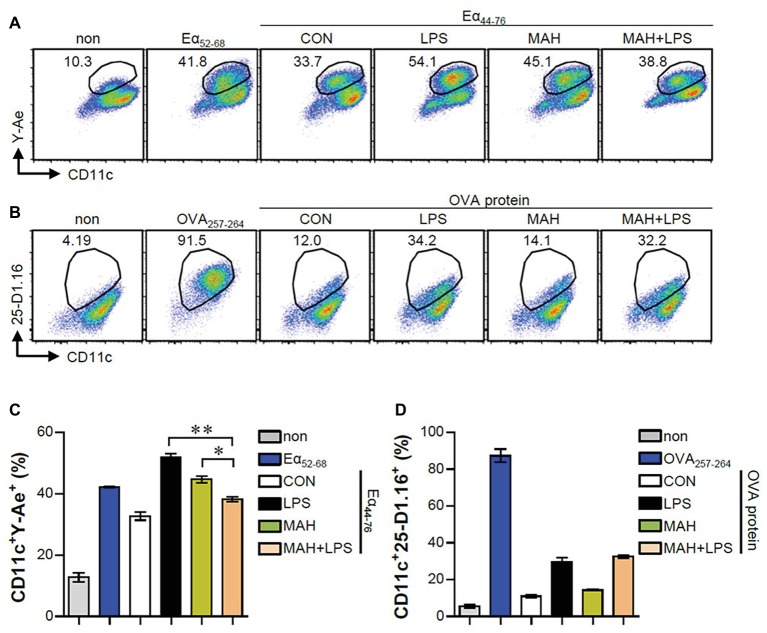
MAH infection reduced the antigen presenting ability of MHC class II molecules in LPS-treated DCs. **(A,C)** DCs were treated with PBS (CON), LPS, MAH, or LPS with MAH, and then exposed to Eα_44–76_ peptide for 24 h. Cells were harvested and stained with ani-CD11c and anti-Y-Ae mAbs. **(B,D)** DCs were treated with PBS (CON), LPS, MAH, or LPS with MAH, and then exposed to OVA protein for 24 h. Cells were harvested and stained with ani-CD11c and anti-25-D1.16 mAbs. Histogram and bar graphs for expression of Eα_52–68_/I-Ab **(A,C)** and OVA_257–264_/H-2Kb **(B,D)** complexes in CD11c^+^ cells were analyzed by flow cytometry. One representative plot out of three independent experiments is shown. ^*^p < 0.05, or ^**^p < 0.01. non: non-treated DCs, Eα_52–68_: Eα_52–68_ peptide-treated DCs (positive control for MHC-II presenting), OVA_257–264_: OVA_257–264_ peptide-treated DCs (positive control for MHC-I presenting).

The IL-10 production level is higher in tolerogenic DCs compared to in inflammatory DCs (Raker et al., 2015). Thus, we hypothesized that IL-10 production by MAH-infected DCs, upon stimulation with LPS, induces tolerogenic phenotypes. To validate this hypothesis, we analyzed the expression of surface molecules (CD80, CD86, MHC-I, and MHC-II) in each stimulation condition (non-, LPS-, MAH-, MAH/LPS-DCs) across the time-points (12 and 24 h). MAH-infected DCs exposed to LPS showed a lower expression of MHC-II than MAH-infected DCs at 24 h, but there was no significant change in CD80, CD86, and MHC-I expression (Figure 2A) at all time-points. These phenomena were not observed at an earlier time point (6 h; data not shown). We also measured the extracellular and intracellular cytokine levels in time-dependent manner. A significant up-regulation of extracellular IL-10 production occurred in MAH-infected DCs exposed to LPS in a time-dependent manner (Figure 2B). These results were confirmed by intracellular staining of cytokines, which revealed that MAH-infected DCs exposed to LPS stimulation exhibited higher intracellular IL-10 expression, but lower intracellular IL-12p70 expression, than LPS-treated DCs (Figure 2C). Since antigen-presenting ability is influenced by the expression of IL-10 and MHC class molecules regulated by DCs, we analyzed the antigen-presenting ability of MAH-infected DCs exposed to LPS signaling by the anti-Y-Ae mAb, which directly reacts with the Eα_52_–_68_ peptide MHC-II, and the anti-25-D1.16 mAb, which recognizes the OVA_257–264_ peptide bound to H-2Kb of MHC-I (Figure 3). In the study of antigen-presenting ability of non-treated DCs, MAH-infected DCs, LPS-treated DCs, and MAH-infected DCs exposed to LPS were treated with OVA protein or Eα_44_–_76_ peptide for 24 h, and then cells were stained with anti-Y-Ae (Figures 3A,C) or anti-25-D1.16 (Figures 3B,D) mAbs, respectively. Eα_52_–_68_ and OVA_257–264_ were used as the positive control for MHC-II or MHC-I bound peptides. As expected, a significantly lower percentage of OVA_257–264_/MHC-II complexes (Y-Ae-positive cells) were present in the MAH-infected DCs exposed to LPS compared to that in the MAH-infected DCs, or LPS-treated DCs (Figures 3A,C); a decrease of Eα_52_–_68_/MHC-I complex (25-D1.16-positive cells) was not observed (Figures 3B,D). These results, together with the strong increase in IL-10 production, reduction of MHC-II expression, and MHC-II antigen presentation, indicated that MAH infection endows DCs with a tolerogenic property in the presence of TLR4 signaling.

### *Mycobacterium avium* subspecies *hominissuis*-Infected Dendritic Cells Reduce the Proliferation of CD4^+^ T Cells in the Presence of Toll-Like Receptor4 Signaling

**Figure 4:**
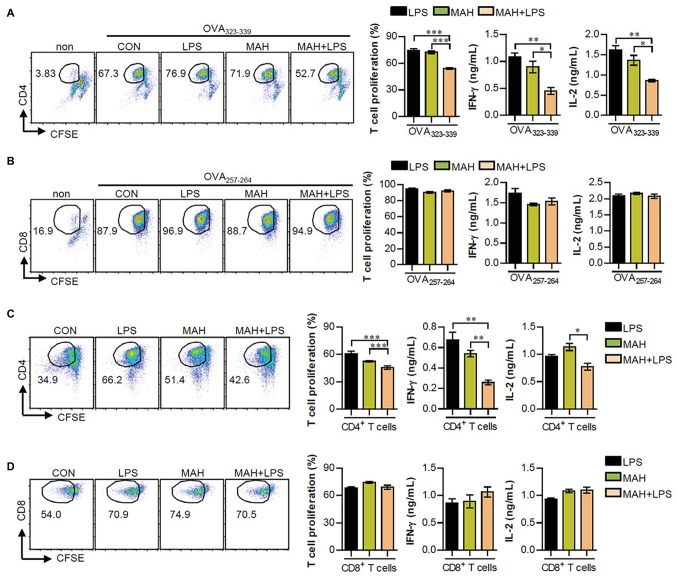
MAH infection reduced the capacity of LPS-activated DCs to induce CD4^+^ T cell proliferation. **(A,B)** OVA-specific T cells (CD4 and CD8) isolated from OT-I and OT-II mice were stained with CFSE, co-cultured for 96 h with DCs treated with PBS (CON), MAH, or LPS with MAH, and then exposed to OVA_323–339_ or OVA_257–264_ peptide. Cells were harvested and stained with ani-CD4 or anti-CD8 mAbs. To analyze T cell proliferation, lymphocyte gate was first drawn based on the cell size and granularity. The proliferated T cells were further gated to obtain CD4^+^CFSE^−^ and CD8^+^CFSE^−^. The proliferation and cytokine production (IFN-γ and IL-2) of OVA-specific CD4^+^ **(A)** and CD8^+^ T cells **(B)** were then assessed by flow cytometry and ELISA, respectively. **(C,D)** CD4 and CD8 T cells isolated from BALB/C (allogeneic mice) were stained with CFSE, and co-cultured for 96 h with DCs treated with PBS (CON), MAH, or LPS with MAH. The proliferation and cytokine production (IFN-γ and IL-2) of CD4^+^ **(C)** and CD8^+^ T cells **(D)** were then assessed by flow cytometry and ELISA, respectively. Dot plots for CD4 and CD8 T cell proliferation are shown as one representative plot out of three independent experiments. Bar graphs are expressed as the mean ± SD of 5 samples per treated condition. IFN-γ and IL-2 production in the culture supernatants were analyzed by ELISA. Bar graphs are expressed as the mean ± SD of 3 samples per treated condition. ^*^p < 0.05, ^**^p < 0.01, or ^***^p < 0.001. non: non-treated DCs.

As a next step, we investigated the reduction of T cell proliferation, induced during MAH-infection of DCs exposed to LPS, which can block the proliferation of naïve T cells (Domogalla et al., 2017). Thus, we performed a mixed lymphocyte reaction assay using OVA-specific T cells (Figures 4A,B) and allogeneic T cells (Figures 4C,D). Further details regarding the mixed lymphocyte reaction assay is provided in the section “Materials and Methods.” Interestingly, MAH-infected DCs exposed to LPS reduced the proliferation and Th1-type cytokine production (IFN-γ and IL-2) of OVA-specific (Figure 4A) and allogeneic (Figure 4C) CD4^+^ T cells compared to MAH-infected DCs and/or LPS-treated DCs, although not in CD8^+^ T cells (Figures 4B,D). These findings indicated that MAH-infected DCs exposed to LPS lead to the inhibitory effect of CD4^+^ T cell proliferation.

### Interleukin-10 Neutralization for *Mycobacterium avium* subspecies *hominissuis*-Infected Dendritic Cells Exposed to LPS Induces Neither Tolerogenic Dendritic Cell Phenotypes nor Reduction in CD4^+^ T Cell Proliferation

**Figure 5:**
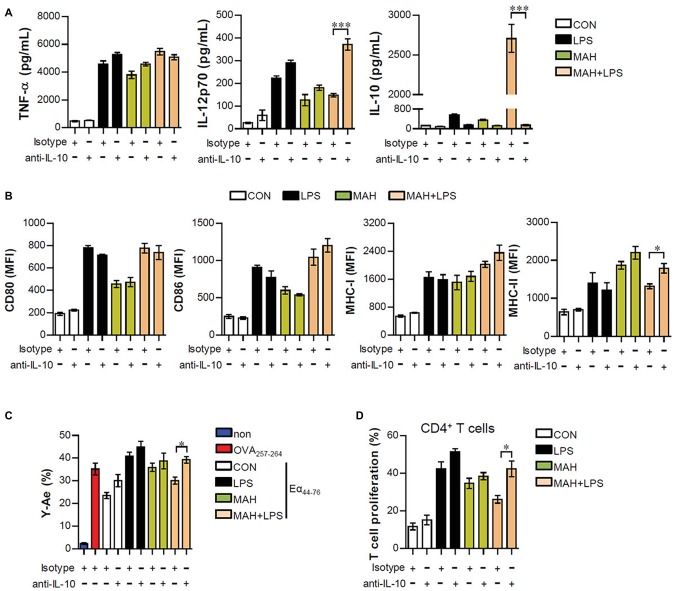
IL-10 neutralization reduced the tolerogenic function induced by MAH/LPS-primed DCs. **(A,B)** DCs were treated with PBS (CON), LPS, MAH, or LPS with MAH and then pulsed with a neutralizing anti-IL-10 mAb or rat IgG (isotype control) for 24 h. **(A)** IL-12p70, TNF-α, and IL-10 production in the culture supernatants were analyzed by ELISA. **(B)** Cells were stained with surface Abs (anti-CD80, anti-CD86, anti-MHC-I, and anti-MHC-II) of DCs and analyzed by flow cytometry. Bar graphs are expressed as the mean ± SD of five samples per treated condition. **(C,D)** In the presence of neutralizing ani-IL-10 mAb or rat IgG, DCs were treated with PBS (CON), LPS, MAH, or LPS with MAH and then exposed to Eα_44–76_ peptide or OVA protein for 24 h. Cells were harvested and stained with ani-CD11c, anti-Y-Ae, or anti-25-D1.16 mAbs. Bar graph for expression of Eα_52–68_/I-Ab complexes was analyzed by flow cytometry. The bar graph is expressed as the mean ± SD of four samples per treated condition. **(D)** CD4^+^ T cells isolated from BALB/C mice (allogeneic mice) were stained with CFSE, and co-cultured for 96 h with DCs treated with PBS (CON), MAH, or LPS with MAH in the presence or absence of a neutralizing ani-IL-10 mAb or rat IgG. The proliferation of CD4^+^ was then assessed by flow cytometry. Bar graph is expressed as the mean ± SD of five samples per treated condition. All data are shown as a representative plot out of three independent experiments. ^*^p < 0.05, or ^***^p < 0.001. non: non-treated DCs.

Based on the above results, we hypothesized that if IL-10 production, induced by MAH-infected DCs exposed to LPS, is a primary factor for inducing tolerogenic DC phenotypes and reducing CD4^+^ T cell proliferation, the absence of IL-10 signaling should restore an activated DC phenotype and CD4^+^ T cell proliferation. First, to investigate whether the IL-10 induced by MAH-infected DCs exposed to LPS induces phenotypic and functional changes, we treated MAH-infected cells with an anti-IL-10 mAb and evaluated cytokine production, surface molecule expression, and antigen-presenting ability exposed to LPS stimulation. MAH-infected DCs exposed to LPS significantly abrogated IL-10 production, although IL-12p70 levels were increased in the presence of anti-IL-10 mAb. TNF-α production was not significantly different in the presence of anti-IL-10 mAb (Figure 5A). Moreover, incubation of the LPS/MAH-infected DCs (MAH-infected DCs exposed to LPS) with an anti-IL-10 mAb restored their MHC-II expression levels (Figure 5B, Supplementary Figure S2A) as well as antigen-presenting ability (Figure 5C, Supplementary Figure S2B) of MHC-II. Furthermore, we evaluated the capacity of IL-10-neutralized LPS/MAH-infected DCs to induce proliferation of allogenic CD4^+^ T cells using the same experimental conditions described in Figure 4C. As shown in Figure 5D, Supplementary Figure S2C, IL-10-neutralized LPS/MAH-infected DCs restored CD4^+^ T cell proliferation compared to rat IgG (isotype control Ab)-treated LPS/MAH-infected DCs. Collectively, these results indicated IL-10 signal as a key player in the tolerogenic phenotypes and activities induced by the MAH-infected DCs exposed to LPS.

### Live *Mycobacterium avium* subspecies *hominissuis* Infection in Dendritic Cells is Essential for Both Toll-Like Receptor2 and Toll-Like Receptor4 Signaling-Mediated Interleukin-10 Production

**Figure 6:**
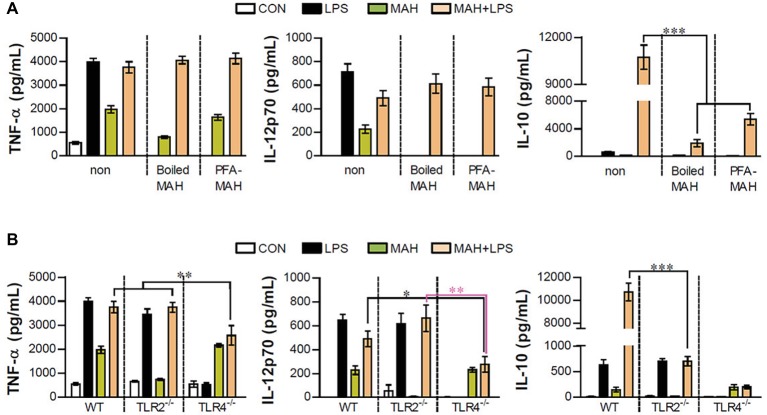
Live MAH induced IL-10 secretion *via* TLR2 receptor. **(A)** Maturation of the MAH vacuole is arrested in DCs. Live- (MAH), PFA fixed- (PFA-MAH), and heat-killed MAH (Boiled MAH)-infected DCs were treated in the presence or absence of LPS for 24 h. Culture supernatants were collected and the levels of TNF-α, IL-12p70, and IL-10 were determined by ELISA assay. **(B)** Bone marrow cells were purified from WT, TLR2^−/−^, and TLR4^−/−^ mice. WT-, TLR2^−/−^-, and TLR4^−/−^-DCs differentiated from each bone marrow cells were treated with PBS (CON), LPS, MAH, or LPS with MAH for 24 h. After that, the secretion of TNF-α, IL-12p70, and IL-10 on DCs was measured by ELISA. Bar graph is expressed as the mean ± SD of four samples per treated condition. All data are shown in one representative plot out of three independent experiments. ^*^p < 0.05, ^**^p < 0.01, or ^***^p < 0.001. non: non-stimulated condition.

Next, we examined whether excessive IL-10 production of LPS/MAH-infected DCs is regulated by live MAH infection, and DCs were exposed to inactivated MAH, killed by heat or PFA treatment, in the presence of LPS. Interestingly, lower levels of IL-10 were observed in the LPS/inactivated MAH-infected DCs than in LPS/MAH-infected DCs (Figure 6A). Moreover, we determined the regulation of IL-10 production in DCs isolated from WT, TLR2^−/−^, and TLR4^−/−^ mice, since TLR2 and TLR4 receptors play critical roles in host immune system against MAH infection (Feng et al., 2003; Bhatnagar and Schorey, 2007). As shown in Figure 6B, the expression of TNF-α, IL-12p70, and IL-10 was induced by MAH-infected DCs exposed to LPS in the same manner as LPS-alone treatment in TLR2^−/−^ and as MAH-alone infection in TLR4^−/−^. However, unlike other cytokines such as TNF-α and IL-12p70, IL-10 production was remarkably increased in the WT. These results suggested that both TLR2 and TLR4 signaling are essential for excessive IL-10 production induced by MAH-infected DCs exposed to LPS.

### Activation of Cox-2 Pathways Mediates Excessive Interleukin-10 Production in *Mycobacterium avium* subspecies *hominissuis*-Infected Dendritic Cells Exposed to LPS

**Figure 7:**
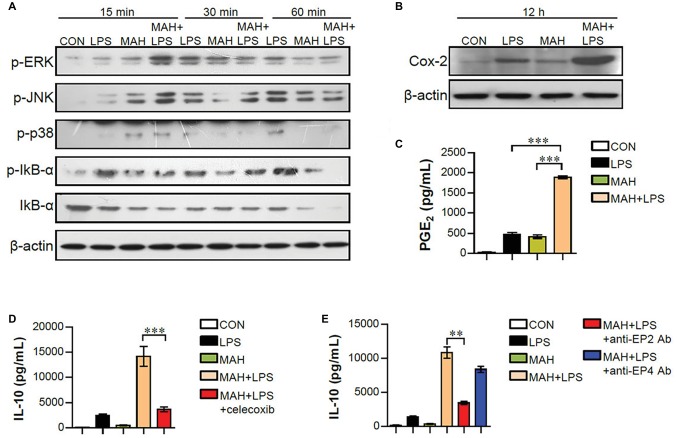
MAH-induced IL-10 production in LPS-treated DCs involved the activation of Cox-2/PGE_2_ signaling pathways. **(A,B)** DCs were treated with PBS (CON), LPS, MAH, or LPS with MAH for indicated times. Protein levels of phospho-ERK (p-ERK), p-JNK, p-p38, p-IkB-α, IkB-α, and COX-2 in cell lysate were evaluated by western blot analysis. β-actin: loading control for cytosolic fractions. **(C)** After stimulation for 24 h, supernatants were collected, and levels of PGE_2_ were determined by ELISA assay. **(D,E)** DCs were cultured at 37°C for 1 h with Cox-2 inhibitor (**D**; celecoxib, 10 μM) or PGE_2_ receptor blockers (**E**; anti-EP2 and -EP4 Abs) for 1 h, prior to treatment with PBS, LPS, MAH, or LPS with MAH for 24 h. Culture supernatants were collected, and IL-10 production analyzed using ELISA. ^***^p < 0.001 or ^**^p < 0.01.

Mitogen-activated protein kinase (MAPK), nuclear factor kappa-B (NF-κB), and cyclooxygenase-2 (Cox2)/PGE_2_ signals regulate pro- and anti-inflammatory cytokine production during DC maturation (Harizi et al., 2002; Gabrysova et al., 2014). To examine whether these signals are involved in increased IL-10 production of LPS/MAH-infected DCs, the phosphorylated MAPKs (p-ERK, p-JNK, and p-p38), the phosphorylation/degradation of IκB-α, and Cox2 activation were investigated by western blot analysis (Figure 7A). LPS/MAH-infected DCs showed rapid ERK, JNK, and p38 phosphorylation compared to LPS-treated or MAH-infected DCs at early exposure time (15 min), but not in later exposure times (30 and 60 min). These results showed that MAPK and NF-κB signals are not the major pathways for IL-10 production, since IL-10 produced by LPS/MAH-infected DCs were not observed in 6-h exposure time. Interestingly, LPS/MAH-infected DCs promoted Cox2 activation and PGE2 expression compared to LPS-treated or MAH-infected DCs (Figures 7B,C). To confirm the involvement of Cox2/PGE2 activation in excessive IL-10 production, we next treated LPS/MAH-infected DCs with specific Cox2 inhibitor (celecoxib) and PGE2 receptor blockers (anti-EP2 and -EP4 mAbs) and then analyzed IL-10 production. Treatment of Cox2 inhibitor and EP2 blocker significantly abrogated IL-10 production in LPS/MAH-infected DCs (Figures 7D,E), suggesting Cox2/PGE2-dependent EP2 signaling as the main pathway for IL-10 production induced by MAH-infected DCs exposed to LPS.

## Discussion

To date, pathogenesis and immune phenomena have been studied using single species of mycobacteria and applied to treatment guidelines; these studies have usually been limited to MTB. Here, we investigated the specific immune response and related major mechanisms by stimulating the different TLR agonists to mimic co-infection in MAH-infected DCs. MAH-infected DCs strongly increased IL-10 production when treated with the TLR4 agonist LPS compared to treatment with various other TLR agonists. LPS treatment induced tolerogenic phenotypes in MAH-infected DCs by significantly reducing the expression of MHC class II and MHC class II-antigen presentation and significantly increasing the production of extracellular and intracellular IL-10, thus inhibiting CD4^+^ T cell proliferation along with decreased IFN-γ and IL-2. The use of an anti-IL-10 neutralizing Ab also confirmed the recovery of all tolerogenic DC phenotypes described above. Interestingly, the expression of these tolerogenic DCs was observed following infection with live MAH, but not with inactivated MAH, and when TLR2 and TLR4 were co-involved. In addition, the tolerogenic phenotype in which MAH-infected DCs were induced by LPS treatment revealed that Cox2/PGE2-dependent EP2 signaling is a key pathway.

As shown in Figure 1A, the expression changes of TNF-α, IL-12p70, and IL-10 following TLR2, TLR3, TLR4, TLR7, and TLR9 agonist treatment in MAH-infected DCs were analyzed. Although there were differences in levels, TLR3 and TLR4 stimulation particularly decreased IL-12p70 expression and remarkably elevated IL-10 expression in MAH-infected DCs. There was a substantial increase in IL-10 expression following TLR4 stimulation (Figure 1C). As mentioned in the section “Introduction,” many studies have suggested *P. aeruginosa*, a Gram-negative organism, as the most frequent co-infecting agent in NTM infection (Wickremasinghe et al., 2005). TLR4 is one of the representative TLRs in *P. aeruginosa*, which has been shown to affect susceptibility phenotypes, such as increased inflammatory cytokine production, decreased iNOs and β-defensin-2 production, and bacterial elimination in TLR4-deficient mice (McIsaac et al., 2012). For further clarification, we identified changes in the expression of TNF-α, IL-12p70, and IL-10 in MAH-infected DCs co-infected with *P. aeruginosa*, and in particular, the expression of IL-10 dramatically increased, confirming that TLR4 signaling in MAH infected cells showed the same result as in the actual infection with Gram-negative bacteria (Supplementary Figure S1). In addition, we analyzed the expression changes of IL-10 production in TLR2^−/-^DCs and TLR4^−/-^DCs following MAH/LPS treatment (Figure 6B). The level of IL-10 expression was similar in that in MAH/LPS-infection and LPS-treatment of TLR2^−/−^ DCs and in MAH/LPS-infection and MAH-infection of TLR4^−/−^ DCs, whereas IL-10 production was strongly increased in MAH/LPS-infection in WT DCs. This level of IL-10 expression was greater than the sum of IL-10 production due to either MAH or LPS alone. These results collectively indicate that MAH infection and LPS stimulation interact and produce a synergistic reaction. TLR2 and TLR4 are thought to act together to express the phenotype of IL-10-expressing tolerogenic DC.

There have been several previous studies in which multiple TLRs have been shown to work together to induce totally new immune responses or to distort the existing immune responses. Along with TLR2, which is a major synergistic receptor that recognizes mycobacteria, TLR9 is essential for inducing Th1 responses (Bafica et al., 2005); however, according to Holscher et al. (2008), TLR2, TLR9, and MyD88 signaling has little influence on the induction of Th1 responses in MTB infection (Holscher et al., 2008). TLR6, TLR9, and MyD88 signaling has been shown to regulate *M. avium* infection; MyD88, in particular, plays an essential role in inducing a protective immune response (Carvalho et al., 2011; Marinho et al., 2013). In addition, Srivastava et al. (2009) had reported that MTB acts on TLR2 and dendritic cell-specific ICAM-3 grabbing non-integrin receptor (DC-SIGNR), and that suppressors of cytokine signaling (SOCS) was induced by the differential control of the two receptors depending on MTB virulence, thus impairing the defense against MTB (Srivastava et al., 2009). More importantly, TLR4,2-primed DCs enhanced IL-10 production by altering the balance of signaling pathways (*via* p38 MAPK, ERK1/2, and TNFR-associated factor 3) following TLR4 stimulation (Yanagawa and Onoe, 2007). MAPK p38 has been well established to be first involved in the production of IL-10 and IL-12 in response to LPS stimulation (Park et al., 2005). However, following TLR4 stimulation, IL-10 production is more significantly increased in TLR4,2-primed DCs than in TLR4-primed DCs, thereby suggesting that TLR2-mediated signaling contributes to the enhancement of IL-10 production capability (Yanagawa and Onoe, 2007). We can see similar results in Figure 6. TNF-α was expressed at the same level regardless of live, inactivated, or dead MAH infection; however, the expression of IL-12p70 and IL-10 was confirmed to be induced during the infection of live MAH (Figure 6A). In addition, since TLR2 and TLR4 were found to work together and boost the expression of IL-10 (Figure 6B), the TLR4 agonist, LPS, and the TLR2 ligand, expressed by live MAH, were thought to induce tolerogenic DC.

DCs are tightly regulated so as to induce protective immune responses and prevent exaggerated or unwanted immune responses (Corinti et al., 2001). IL-10 is an anti-inflammatory cytokine and a critical factor in inducing tolerogenic DCs, which subsequently impedes the Th1 response (Raker et al., 2015; Kim et al., 2017). The role of IL-10 in mycobacterial infection has been extensively studied. IL-10 has been shown to play an important role in immune regulation in host immunity; however, it is also known to be involved in anti-mycobacterial function, which can survive in both macrophages and DCs (Hussain et al., 2016). In the present study, the high level of IL-10 produced in LPS/MAH-infected DCs selectively promoted tolerogenic DCs by reducing MHC class II and MHC class II-antigen presentation and consequently inhibiting CD4^+^ T cell proliferation and IFN-γ and IL-2 expression (Figures 2–5). Finally, IL-12p70 expression was reduced in LPS/MAH-infected DCs, while IL-12p70 expression was restored using anti-IL-10, demonstrating that Th1 responses were inhibited by LPS/MAH stimulation (Figure 5). These findings clearly imply that the tolerogenic phenotype of DCs was induced by IL-10.

Various mechanisms have been reported to modulate IL-10 production. The major signaling pathways associated with IL-10 regulation and production include MAPKs, NF-κB, and signal transducer and activator of transcription-3 (STAT3) (Hussain et al., 2016). In particular, MAPK p38 plays an important role in overall signal cascades in mycobacterial infections (Reiling et al., 2001; Hussain et al., 2016). However, in this study, since IL-10 was produced after 6 h of LPS stimulation and MAPK (ERK, JNK, and p. 38) phosphorylation only occurred during the early exposure time (15 min), the MAPK signals were obviously not the major pathway involved in IL-10 production (Figure 7). The adapter MyD88, which acts downstream of TLR4, induces activation of NF-κB and MAP kinase using the adapter TIRAP (Takeuchi and Akira, 2010); TIR-domain-containing adapter-inducing interferon-β (TRIF) activates IRF3 as an adapter for TLR4 and TLR3 and induces expression of IFN-β and -α4 (Takeuchi and Akira, 2010). As mentioned above, TLR3 and TLR4 treatment of MAH-infected DCs showed similar results, indicating increased IL-10 expression and decreased IL-12p70 expression. Although previous studies have reported differences in TRIF-mediated DC maturation for type 1 IFN dependencies between TLR3 and TLR4 (Hu et al., 2015), our results indicated the involvement of TRIF signaling pathway downstream of TLR3 and TLR4.

In addition to IL-10, PGE2 is also produced in DCs and is known to exhibit immunosuppressive effects (Harizi and Gualde, 2006). Moreover, PGE2 has been reported to act on the EP receptor, a PGE2 receptor expressed on the surface of DCs, to increase internal cAMP, which ultimately increases endogenous IL-10 production (Harizi and Norbert, 2004). Cox2 has been reported to be highly expressed in DCs following LPS stimulation and is associated with the synthesis of large amounts of PGE2 (Harizi et al., 2002). Interestingly, Cox2 activation and PGE2 expression were significantly increased in LPS/MAH-infected DCs compared to LPS-treated or MAH-infected DC alone, and the production of IL-10 was remarkably inhibited by celecoxib, a Cox2 inhibitor, in the present study (Figure 7). Therefore, IL-10 expression was considered to be induced by Cox2-mediated PGE2 in MAH/LPS infected DCs. Of the four subtypes, cAMP has been reported to act on EP2 and EP4, IL-10 is increased in DCs, and EP2 increases IL-10 production in LPS-stimulated DCs (Harizi et al., 2003; Harizi and Gualde, 2006). Our results demonstrate the effect of EP2 blocker in reducing IL-10 production, thereby suggesting Cox2/PGE2-dependent EP2 signaling as the major mechanism involved in IL-10 production induction by LPS treatment in MAH-infected DCs. Therefore, the present study on tolerogenic DC phenotype, function, and related mechanisms induced by LPS/MAH-infected DCs may provide clues for understanding the altered immune responses in case of MAH co-infection.

## Data Availability

All relevant datasets are contained within the manuscript.

## Ethics Statement

This study was carried out in accordance with the regulation of “the Institutional Animal Care and Use Committee of the Yonsei University Health System” (Permit number: 2015-0203). The protocol was approved by “the Institutional Animal Care and Use Committee of the Yonsei University Health System.”

## Author Contributions

WK performed most of the experiments. WK, J-HY, M-KS, and SS conceived the study, analyzed the data, and wrote the manuscript. M-KS and SS critically revised the manuscript. All the authors discussed the results and commented on the manuscript.

### Conflict of Interest Statement

The authors declare that the research was conducted in the absence of any commercial or financial relationships that could be construed as a potential conflict of interest.

## Supplementary Material

SUPPLEMENTARY FIGURE 1 MAH-infected DCs induced high-level anti-inflammatory cytokine secretion during *Pseudomonas aeruginosa* infection. Immature DCs were treated with PBS (CON), MAH (at a MOI of 1), *P. aeruginosa* PA01 (at a MOI of 0.1, 0.5, and 1), *P. aeruginosa* NCCP14781 (at a MOI of 0.1, 0.5, and 1), or *P. aeruginosa* with MAH. After 24 h, culture supernatants were collected, and TNF-α, IL-12p70, and IL-10 productions were analyzed using ELISA. The data are expressed as the mean ± SD of 4 samples per treated condition. ^***^*p* < 0.001.

SUPPLEMENTARY FIGURE 2 Gating strategy for the analysis of surface molecule expression, antigen presenting ability, and T cell proliferation by IL-10 neutralization in DCs treated with LPS and MAH. **(A,B)** DCs were treated with PBS (CON), LPS, MAH, or LPS with MAH, and then pulsed with a neutralizing ani-IL-10 mAb or rat IgG (isotype control) for 24 h. **(A)** Cells were stained with surface Abs (anti-CD80, anti-CD86, anti-MHC-I, anti-MHC-II) of DCs, and analyzed by flow cytometry. This result shows the flow cytometry gating strategy for Figure 5B. **(B)** In presence of neutralizing ani-IL-10 mAb or rat IgG, DCs were treated with PBS (CON), LPS, MAH, or LPS with MAH, and then exposed to Eα_44–76_ peptide or OVA_257–264_ (positive control) for 24 h. Cells were harvested and stained with ani-CD11c, and anti-Y-Ae or anti-25-D1.16 mAbs. Bar graph for the expression of Eα_52–68_/I-Ab complexes was derived from flow cytometry. This result shows the flow cytometry gating strategy for Figure 5C. **(C)** CD4^+^ T cells isolated from BALB/C (allogeneic mice) were stained with CFSE, and co-cultured for 96 h with DCs treated with PBS (CON), MAH, or LPS with MAH, in presence or absence of a neutralizing ani-IL-10 mAb or rat IgG. The proliferation of CD4^+^ was then assessed by flow cytometry. This result shows the flow cytometry gating strategy for Figure 5D.
