# Supplementary figures and images for: Infection of Dendritic Cells With Mycobacterium avium subspecies hominissuis Exhibits a Functionally Tolerogenic Phenotype in Response to Toll-Like Receptor Agonists via IL-10/Cox2/PGE2/EP2 Axis
Source: Front Microbiol. 2019 Aug 7;10:1795. doi: 10.3389/fmicb.2019.01795 (PMC6692481; doi:10.3389/fmicb.2019.01795)

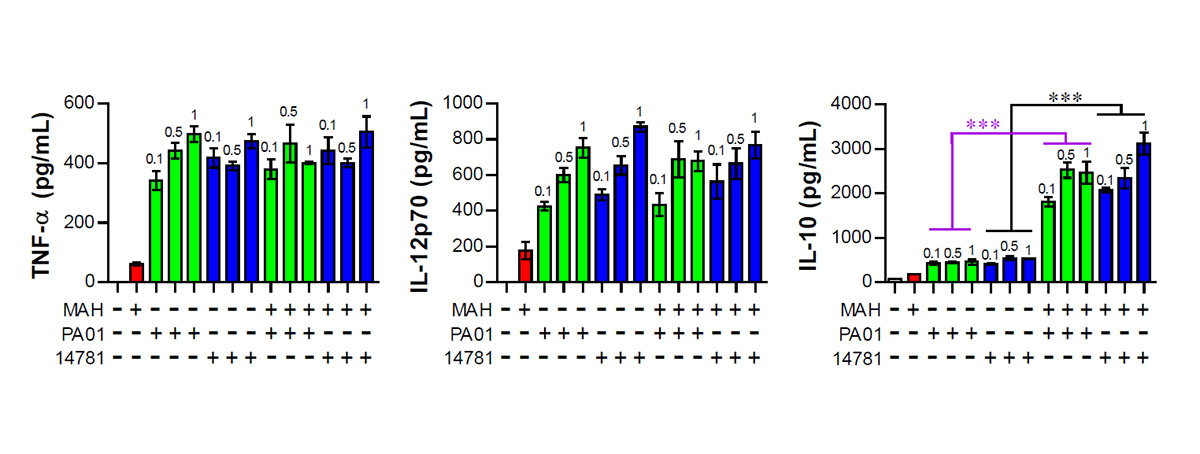

Supplement: SUPPLEMENTARY FIGURE 1 — MAH-infected DCs induced high-level anti-inflammatory cytokine secretion during Pseudomonas aeruginosa infection. Immature DCs were treated with PBS (CON), MAH (at a MOI of 1), P. aeruginosa PA01 (at a MOI of 0.1, 0.5, and 1), P. aeruginosa NCCP14781 (at a MOI of 0.1, 0.5, and 1), or P. aeruginosa with MAH. After 24 h, culture supernatants were collected, and TNF-α, IL-12p70, and IL-10 productions were analyzed using ELISA. The data are expressed as the mean ± SD of 4 samples per treated condition. ***p < 0.001. [file Image_1.jpg]

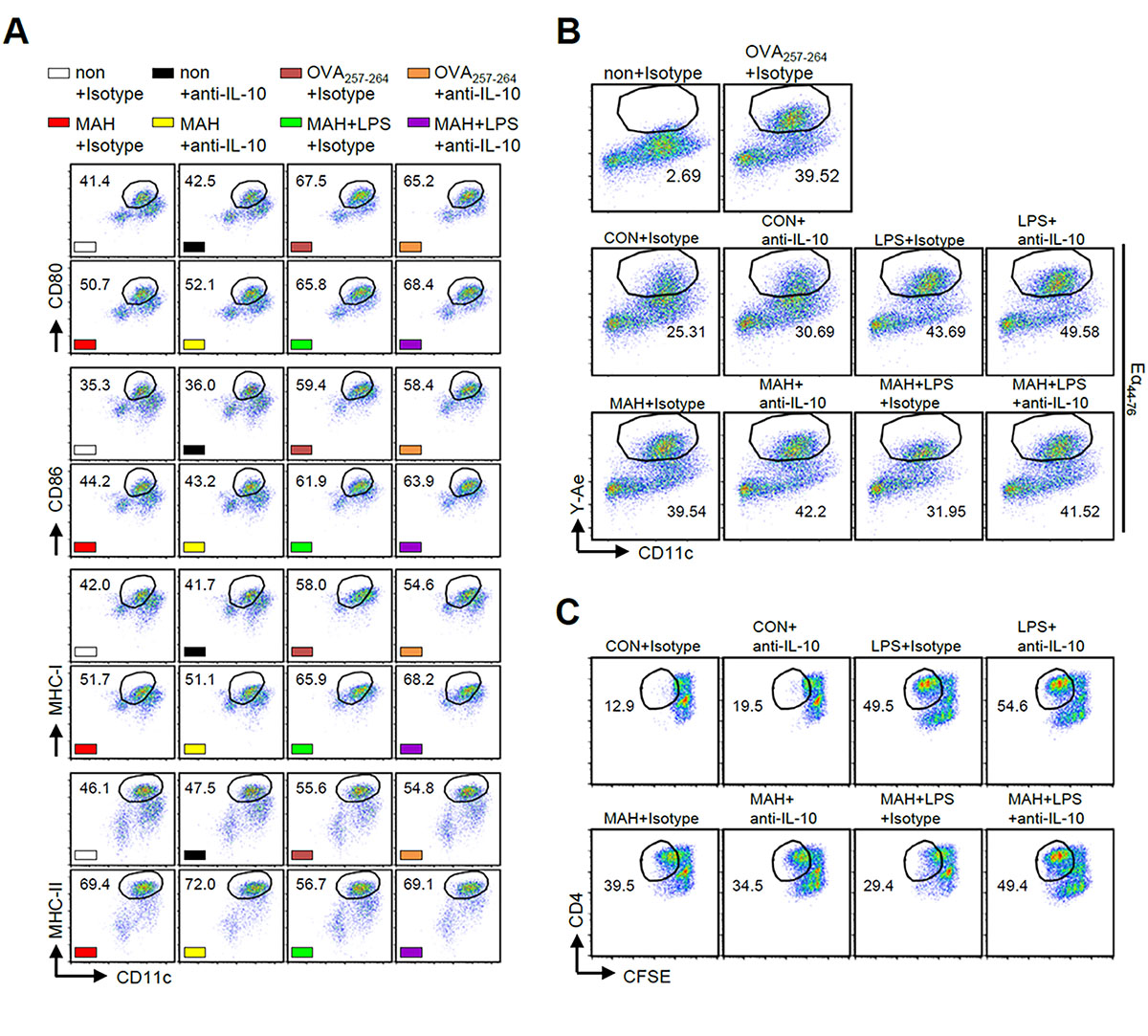

Supplement: SUPPLEMENTARY FIGURE 2 — Gating strategy for the analysis of surface molecule expression, antigen presenting ability, and T cell proliferation by IL-10 neutralization in DCs treated with LPS and MAH. (A,B) DCs were treated with PBS (CON), LPS, MAH, or LPS with MAH, and then pulsed with a neutralizing ani-IL-10 mAb or rat IgG (isotype control) for 24 h. (A) Cells were stained with surface Abs (anti-CD80, anti-CD86, anti-MHC-I, anti-MHC-II) of DCs, and analyzed by flow cytometry. This result shows the flow cytometry gating strategy for Figure 5B. (B) In presence of neutralizing ani-IL-10 mAb or rat IgG, DCs were treated with PBS (CON), LPS, MAH, or LPS with MAH, and then exposed to Eα44–76 peptide or OVA257–264 (positive control) for 24 h. Cells were harvested and stained with ani-CD11c, and anti-Y-Ae or anti-25-D1.16 mAbs. Bar graph for the expression of Eα52–68/I-Ab complexes was derived from flow cytometry. This result shows the flow cytometry gating strategy for Figure 5C. (C) CD4+ T cells isolated from BALB/C (allogeneic mice) were stained with CFSE, and co-cultured for 96 h with DCs treated with PBS (CON), MAH, or LPS with MAH, in presence or absence of a neutralizing ani-IL-10 mAb or rat IgG. The proliferation of CD4+ was then assessed by flow cytometry. This result shows the flow cytometry gating strategy for Figure 5D. [file Image_2.jpg]
